# Oxidation of Peroxiredoxin 6 in the Presence of GSH Increases its Phospholipase A_2_ Activity at Cytoplasmic pH

**DOI:** 10.3390/antiox8010004

**Published:** 2018-12-24

**Authors:** Suiping Zhou, Chandra Dodia, Sheldon I. Feinstein, Sandra Harper, Henry J. Forman, David W. Speicher, Aron B. Fisher

**Affiliations:** 1 Institute for Environmental Medicine, University of Pennsylvania Perelman School of Medicine, Philadelphia, PA 19104, USA; Suiping.Zhou@STJUDE.ORG (S.Z.); cdodia@mail.med.upenn.edu (C.D.); sif@pennmedicine.upenn.edu (S.I.F.); 2 Center for Systems and Computational Biology, The Wistar Institute, Philadelphia, PA 19104, USA; sharper@Wistar.org (S.H.); speicher@wistar.org (D.W.S.); 3 Leonard Davis School of Gerontology, The University of Southern California, Los Angeles, CA 19104, USA; peroxideman@gmail.com

**Keywords:** substrate binding, sulfinic acid, Prdx6 structure, mass spectroscopic analysis

## Abstract

The expression of the phospholipase A_2_ activity (aiPLA_2_) of peroxiredoxin 6 (Prdx6) in the cell cytoplasm is physiologically relevant for the repair of peroxidized cell membranes, but aiPLA_2_ assay *in vitro* indicates that, unlike assay at pH 4, activity at cytosolic pH is essentially absent with non-oxidized substrate. However, the addition of glutathione (GSH) to the assay medium significantly increased aiPLA_2_ activity at cytosolic pH, while oxidized GSH (GSSG) and several other thiols had no effect. By mass spectroscopy (ESI MS), the addition of GSH to Prdx6 paradoxically led to oxidation of its conserved Cys47 residue to a sulfinic acid. The effect of GSH on PLA_2_ activity was abolished by incubation under anaerobic conditions, confirming that auto-oxidation of the protein was the mechanism for the GSH effect. Analysis by circular dichroism (CD) and tryptophan fluorescence showed alterations of the protein structure in the presence of GSH. Independently of GSH, the oxidation of Prdx6 by exposure to H_2_O_2_ or the presence of oxidized phospholipid as substrate also significantly increased aiPLA_2_ activity at pH 7. We conclude that the oxidation of the peroxidatically active Cys47 of Prdx6 results in an increase of aiPLA_2_ activity at pH 7 without effect on the activity of the enzyme at pH 4.

## 1. Introduction

Peroxiredoxins (Prdxs) are a widely distributed family of antioxidant enzymes that use a cysteine thiol group as a catalytic center to catalyze the reduction of hydroperoxides [1]. These enzymes function in anti-oxidant defense and they also have an important role in cell signaling through the regulation or sensing of local peroxide concentration [2]. The mammalian peroxiredoxin (Prdx) family consists of six members with a common mechanism for their peroxidase activity. The three-step peroxidatic cycle for the Prdx proteins involves: (i) reduction of a hydroperoxide substrate through the oxidation of a catalytic Cys to a sulfenic acid, followed by; (ii) formation of a disulfide; and, (iii) reduction of the disulfide to regenerate the active protein. The last mammalian peroxiredoxin to be described was Prdx6, a protein that is expressed in essentially all tissues but at particularly high levels in lung, brain, eye, and testes [3,4,5]. While Prdx6 is similar to Prdxs 1–5 in its use of a catalytic Cys residue (C47 in Prdx6) to reduce H_2_O_2_, short chain hydroperoxides, and peroxynitrite (step i above) [6,7], it expresses several unique features that distinguish it from other Prdx family members.

A major difference between Prdx6 and Prdxs 1–5 relates to both steps (ii) and (iii) (the resolution phases) of the peroxidatic catalytic cycle. These steps in the cycle are accomplished in Prdxs 1–5 by a Cys that is intrinsic to the protein (called the resolving Cys), followed by reduction of the disulfide with thioredoxin [3]. On the other hand, Prdx6 uses glutathione (GSH) catalyzed by GSH S-transferase (GST) to complete the reaction cycle [8,9,10]. Based on the characteristics of the catalytic mechanism reflecting the number of conserved cysteine residues, Prdxs 1–5 have been called 2-Cys enzymes. While Prdx6 has been called 1-Cys Prdx. Another important difference between Prdx6 and other family members is that Prdx6, unlike Prdx 1–5, has the ability to reduce phospholipid hydroperoxides with a rate constant similar to that for the reduction of H_2_O_2_ (∼10^6^ M^−1^ s^−1^) [9,11]. 

In addition to differences in the peroxidatic reaction cycle and importantly for this manuscript, Prdx6 expresses several enzymatic functions that are not expressed by Prdxs 1–5. One of these enzymatic functions of Prdx6 is a phospholipase A_2_ (PLA_2_) activity. This latter activity is not associated with C47 but is based on an enzymatically active site consisting of a Ser-His-Asp catalytic triad [12]. Finally, Prdx6 has a third enzymatic activity that is based on a HxxxxD active site (spanning the amino acids 26–31) that catalyzes lysophosphatidylcholine acyl transferase activity (LPCAT) [13]. Thus, Prdx6 appears to play a unique role in phospholipid metabolism with the ability to catabolize (PLA_2_ activity) and/or remodel phospholipids (LPCAT activity) as well as to maintain these phospholipids in their reduced state (peroxidase activity). Regulation of the PLA_2_ activity of Prdx6 is the major focus of this mms. 

Our initial reports describing Prdx6-PLA_2_ activity indicated that the enzyme is active at acidic pH (pH 4), but it has relatively little activity at neutral pH and above [8]. This enzymatic activity at acidic pH is consistent with the localization of the protein to acidic organelles, such as lung lamellar bodies and lysosomes, where it plays an important role in phospholipid turnover [6,14]. Thus, when first described, the enzyme was given the trivial name acidic, Ca^2+^ independent PLA_2_ (aiPLA_2_) to reflect its acidic pH requirement for catalysis and its catalytic activity in the absence of Ca^2+^ [15,16]. However, subsequent studies have indicated that the PLA_2_ activity of Prdx6 also plays important roles in the repair of peroxidized cell membranes [17,18], as well as in the activation of NADPH oxidase (type 2) [19,20], functions that presumably require PLA_2_ activity in the cytosol at approximately neutral pH. This requirement can be accomplished by several mechanisms. First, phosphorylation of the protein results in a markedly increased activity at pH 7–8 (as well as at acidic pH) [21]; we have shown that conformational change of Prdx6 upon its phosphorylation is the basis for the enhancement of substrate binding and increased PLA_2_ enzymatic activity [22]. Second, the presence of an oxidized substrate, e.g., a phospholipid hydroperoxide, significantly enhanced enzymatic activity at neutral pH [23]. As a third mechanism, oxidation of the conserved C47 of Prdx6 following the treatment of cells with H_2_O_2_ resulted in increased PLA_2_ activity at neutral pH [24]. 

An additional mechanism that results in increased Prdx6-PLA_2_ activity at pH 7 was discovered by serendipity. As described above, GSH is required for Prdx6 peroxidase activity; it is commonly used as a reactant in a peroxidase activity assay that is routinely carried out at pH 7 [9]. During our use of this assay, we identified products in the incubation medium that appeared to reflect the presence of PLA_2_ activity and postulated that this might have been due to the presence of GSH. Further study indicated that GSH did indeed stimulate Prdx6-PLA_2_ activity at pH 7, while there was no effect on activity at pH 4 [25]. The present study was undertaken to document this result and to evaluate a possible mechanism for this effect of GSH. This publication is part of a forum on Peroxiredoxin 6 as a Unique Member of the Peroxiredoxin Family.

## 2. Materials and Methods

### 2.1. Reagents

1,2-Bis palmitoyl-*sn*-glycero-3-phosphocholine (DPPC), egg yolk phosphatidylcholine (PC), phosphatidylglycerol (PG), 1-palmitoyl-2-linoleoyl-*sn*-glycero-3-phosphocholine (PLPC), phosphatidylserine (PS), and cholesterol (chol) were purchased from Avanti-Polar Lipids (Birmingham, AL). Extracellular-signal-regulated kinase (Erk2) was purchased from Upstate (Millipore, Billerica, MA, USA). Protein concentration was measured by Coomassie blue binding using bovine γ-globulin as the standard (Bio-Rad, Hercules, CA, USA). A mouse monoclonal antibody against human Prdx6 was purchased from Chemicon EMD Millipore (Billerica, MA, USA). Bromoenol lactone (BEL) was obtained from Cayman Chemical (Ann Arbor, MI, USA). Reduced glutathione (GSH), oxidized glutathione (GSSG), MJ33 (1-hexadecyl- 3-trifluoroethylglycero -*sn*-2-phosphomethanol), tris (2-carboxyethyl) phosphine (TCEP), dithiothreitol (DTT), and all other chemicals were purchased from Sigma-Aldrich (St. Louis, MO, USA). 

### 2.2. Production of Recombinant Prdx6

The expression of human codon optimized Prdx6 plasmid pJexpress 414:75271—prdx6-optEc in Escherichia coli BL21 (DE3) (Novagen, Madison, WI, USA) has been described previously [25]. The purification and identification of recombinant human Prdx6 protein was carried out by the modification of previously described methods for isolation of the rat and human proteins [10,22,23,26]. Purification of recombinant Prdx6 by chromatography utilized an ion exchange diethylaminoethyl (DEAE) column in a fast protein liquid chromatography (FPLC) AKTA purifier system controlled by Unicon 5.1 software (GE Healthcare Biosciences, Uppsala, Sweden). The column was equilibrated with 40 mM Na-acetate buffer (pH 5) containing 1 mM ethylenediaminetetraacetic acid (EDTA), 3% glycerol, and 1 mM TCEP. Since the isoelectric point for Prdx6 is ∼6.0, it does not bind to positively charged DEAE resin at pH 5 and the Prdx6 protein was collected from the flow-through. (The column was rejuvenated by eluting bound (non-Prdx6) proteins using a high salt solution (40 mM Na-acetate, 2 M NaCl, 1 mM EDTA, 3% glycerol, 1 mM TCEP, pH 5) (see Supplemental Figure S1). The purified Prdx6 fraction was concentrated using a 10 kDa molecular mass cut-off Amicon Ultra filter (Millipore, Billerica, MA, USA) followed by dialysis against 50 mM Tris-HCl buffer (pH 8) containing 1 mM EDTA, 3% glycerol, and 1 mM TCEP with a 10 kDa molecular mass cut-off Slide-A-Lyzer® dialysis cassette (Pierce, Rockford, IL, USA). The preparation was evaluated by polyacrylamide gel electrophoresis (PAGE) stained with Coomassie blue (see Supplemental Figure S1). Immunoblots with monoclonal anti-Prdx6 as the primary and goat anti-mouse IgG as the secondary antibody were used to confirm the presence of Prdx6 in the purified protein preparation; the blots were analyzed and the purity in each band was calculated by the two-color Odyssey technique (LI-COR, Lincoln, NE, USA) [26]. The protein was stored at −80 °C before use. For some studies, Prdx6 was phosphorylated by incubation with ERK2 in the presence of ATP and MgCl_2_, as described previously [22] or oxidized by treating the purified protein with 100 µM H_2_O_2_ for 15 min.

### 2.3. Measurement of aiPLA_2_ Activity

The measurement of Prdx6-PLA_2_ (aiPLA_2_) activity has been described previously [4,12,15,16]. A liposomal preparation of lipids reflecting the composition of lung surfactant was used as the substrate for the assay. The lipid mixture contained DPPC, PC, Chol, and PG in the molar ratio 50:25:15:10; liposomes were labeled with tracer [^3^H-9,10-palmitate]-DPPC in the *sn*-2 position. This substrate was used for all assays, unless a different substrate is specifically indicated. Lipids that were dissolved in chloroform were evaporated to dryness under N_2_ onto the wall of a Corex glass centrifuge tube; the evaporated film was resuspended in 50 mM Tris-HCl in phosphate-buffered saline (PBS), pH 7.4, vigorously mixed, frozen, and thawed three times by alternating liquid N_2_ and a 50 °C water bath, and then extruded under pressure at 50 °C for 10 cycles through a 0.1 μm pore size polycarbonate filter. Recovery of ^3^H in the liposome preparation was >95% of the original disintegrations per min (DPMs) that were added to the lipid mixture. Analysis with a dynamic light scattering (DLS) 90 Plus Particle size Analyzer (Brookhaven Instruments, Holtsville, NY, USA) showed a homogeneous population of unilamellar vesicles with a diameter of 100–120 nm. Liposomes were stored overnight at 4 °C before use. 

In experiments, to study the role of an oxidized phospholipid substrate, oxidizable liposomes were generated with PLPC substituting for egg PC in the standard liposomes. These liposomes were exposed to an ^•^OH-generating system (10 μM Cu^2+^ in the presence of 0.2 mM ascorbate) [27] for 45 min at room temperature. The liposomes were dialyzed for 2 h against PBS (pH 7.4) using a Slide-A-Lyzer® dialysis cassette as above. These liposomes presumably contained PLPCOOH as an oxidized lipid component.

To measure PLA_2_ activity, enzyme (Prdx6) in buffer (Tris-EGTA, pH 7.4) was pre-incubated for 15 min and the reaction was started by addition of substrate (^3^H-labeled liposomes) for a 1 h incubation. Lipids were then extracted from the liposomal suspension, separated by thin layer chromatography (TLC), and the free fatty acid band was analyzed by scintillation counting. PLA_2_ activity was calculated from the liberation of ^3^H-palmitic acid.

To measure PLA_2_ activity under anaerobic conditions, the incubation medium first was purged of air in an N-EVAP (Organomation Assoc., Northborough, MA, USA) using a continuous flow of 100% N_2_ for 10 min; N_2_ flow then was maintained during the pre-incubation and incubation periods.

### 2.4. Mass Spectroscopy (ESI- MS and LC-MS/MS)

Both hPrdx6 WT and hPrdx6 C91S were gel-filtered using two Superdex 75 (GE Healthcare, Chicago, IL, USA) columns that were connected in series and equilibrated in 10 mM Tris, 100 mM NaCl, pH 7.4 (TBS), and maintained at 4 °C. Each sample was diluted to 0.55 mg/mL in TBS, with or without addition of 5 mM GSH (final concentration), pH 7.4. Samples were incubated for 1 h at 37 °C, and then stored at 0 °C. After 16–20 h of storage, TCEP (5 mM final concentration) was added to half of each sample. The samples were incubated for 30 min at 37 °C and then stored on ice until analysis by electrospray ionization mass spectrometry (ESI MS). Prior to injection onto the liquid chromatography-mass spectroscopy (LC-MS) system, samples were diluted to 0.1 mg/mL. A Dionex C4 Trap column and a self packed Poros C8 analytical column (20 cm) were interfaced directly with an Orbitrap XL operating at 100 K resolution. Thermo Xtract software (Thermo Fisher Scientific, Waltham, MA, USA) was used for deconvolution of the monoisotopic masses. A portion of each sample was alkylated with iodoacetamide, followed by denaturation with urea, reduction with DTT, and then alkylation with acrylamide. Samples were digested with trypsin and analyzed using liquid chromatography-tandem mass spectrometry (LC-MS/MS) using an LTQ-Orbitrap XL™ mass spectrometer (Thermo Fisher Scientific Waltham, MA, USA). Data was searched using MaxQuant software (Max Planck Institute of biochemistry, München, Germay). The following cysteine mass modifications were included in the search: iodoacetamide derivative, 57.0215 Da; acrylamide adduct, 71.0371 Da; 2 oxidations, 31.9898 Da; 3 oxidations, 47.9847 Da. The intensity value from the MaxQuant output files for the variable modified cysteine containing peptides DFTPVCTTELGR (C47) and DINAYNCEEPTEK (C91) were plotted with the Excel program to show the relative quantitation for each peptide that was obtained after treatment of the protein with the different buffer conditions. 

### 2.5. Fluorescence Spectroscopy 

Fluorescence spectroscopy was performed with a spectrofluorometer (PTI, Photon Technology International, Lawrenceville, NJ, USA) equipped with a water bath temperature-controlled sample holder, a single-photon counting system for fluorescence intensity detection, and dual fluorescence and absorbance channels. Measurements were performed with 1 μM protein at 22 °C in microquartz fluorescence cuvettes with a path length of 0.3 cm. Fluorescence was excited at 295 nm and recorded in the range of 310–450 nm (the emission spectrum of tryptophan) with a 1 nm slit width for both excitation and emission.

### 2.6. Circular Dichroism (CD) 

Prdx6 (6.5 μM in 10 mM Tris buffer, pH 7) was analyzed for CD in a fused quartz cell with a path length of 0.1 cm using a Chirascan™ CD Spectrometer (Applied Photophysics Ltd. Surrey, UK). Protein with or without GSH or GSSG (5 mM) was incubated for 1 h at room temperature in 10 mM Tris buffer, pH 7. Spectra were recorded with three repeats in the far-ultraviolet region (190–260 nm) with a bandwidth of 1.0 nm, a step size of 0.5 nm, and an integration time of 30 s.

### 2.7. Statistical Analysis

Data are expressed as means ± standard error (SE). Differences between mean values were analyzed by two-tailed Student’s *t* test and they were considered statistically significant at *p* < 0.05.

## 3. Results

### 3.1. Effect of GSH on the PLA_2_ Activity of Prdx6 

**Figure 1:**
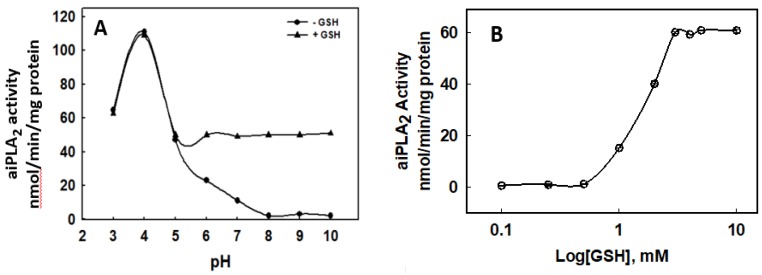
Glutathione (GSH) stimulates the PLA2 activity of Prdx6. (**A**). pH-dependence for the effect of GSH. GSH concentration was 5 mM. (**B**). Effect of GSH concentration on the stimulation of PLA_2_ activity. The effect of GSH is maximal at 3 mM.

The PLA_2_ activity of recombinant human Prdx6 measured at pH 4 was ~100 nmol/min/mg protein (not shown), but it was <1 nmol/min/mg protein when measured at pH 7.4 using the standard liposomal (non-oxidized) substrate described in Methods (Table 1). The addition of GSH (5 mM) to the incubation medium had no effect on aiPLA_2_ activity at pH 4 (not shown) but increased activity at pH 7.4 to ~50% of the pH 4 value (Table 1). The stimulation of aiPLA_2_ activity by GSH was seen at pH > 5 (Figure 1). Stimulation of activity was dependent on GSH concentration with maximal effect at 3 mM GSH (Figure 1). GSH-stimulated activity was sensitive to MJ33, a known inhibitor of aiPLA_2_ activity, while BEL, an inhibitor of some intracellular, Ca^2+^ independent PLA_2_ enzymes, was ineffective (Table 1). The addition of oxidized GSH (GSSG) or the unrelated thiols, DTT, or TCEP, to the assay resulted in a relatively minor increase in aiPLA_2_ activity that was not statistically different from control (Table 1). 

The basal activity and stimulation of activity by GSH at pH 7.4 was similar for control liposomes (DPPC as substrate) and liposomes containing linoleoyl in the *sn*-2 position of PC (PLPC) as the substrate for the PLA_2_ reaction (Table 1). As reported previously, aiPLA_2_ activity at pH 7.4 with oxidized PLPC (i.e., PLPCOOH) as the liposomal substrate was significantly increased when compared to control [17,23] and it was increased to a much greater extent with the phosphorylation of Prdx6 [21,22]. Oxidation of the Prdx6 protein increased its aiPLA_2_ activity with reduced substrate to the same level as with oxidized liposomes. Activity with PLPCOOH substrate, phosphorylated Prdx6, or with oxidized Prdx6 was not increased further in the presence of GSH (Table 1). 

### 3.2. Molecular O_2_ is Required for Generation of the Sulfinic Prdx6

We postulated that the generation of the sulfinic form of Prdx6 in the presence of GSH represented auto-oxidation of the sulfenic form of the protein. To test this hypothesis, the assay for aiPLA_2_ activity was carried out under anaerobic conditions. The aiPLA_2_ activity of the protein was markedly inhibited in the absence of O_2_ (Table 1). The failure of aiPLA_2_ activity to increase in the presence of GSH under anaerobic conditions is compatible with a requirement of molecular oxygen for the oxidation of the sulfenic Prdx6. It is likely that the actual oxidant under aerobic conditions is H_2_O_2_, generated from molecular O_2_ in the presence of trace metals [28].

### 3.3. Prdx6 Modifications in the Presence of GSH Evaluated by ESI- MS and LC-MS/MS

**Figure 2:**
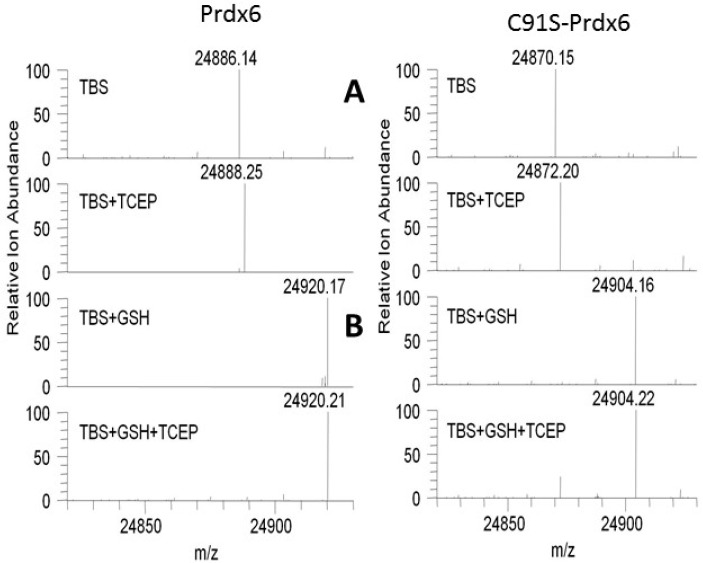
Electrospray ionization mass spectrometry (ESI MS) of wild type and C91S Prdx6. (**A**) upper 4 panels). Effect of tris (2-carboxyethyl) phosphine (TCEP) on protein mass. The addition of TCEP resulted in an increase of 2 Da in molecular mass of both wild type and C47S-Prdx6. (**B**) lower 4 panels). Effect of glutathione (GSH) +/- TCEP on protein mass. The addition of GSH resulted in a mass increase of 34 Da; there was no further effect with addition of TCEP in the presence of GSH. TBS. tris-buffered saline. **A** and **B** were part of the same experiment; **A**, but not **B**, was published previously [26] and it is reprinted with permission.

The effect of GSH on the mass of Prdx6 as well as C91S-Prdx6 was determined using ESI MS (Figure 2). C91 is a non-conserved Cys residue that is present in the human-derived Prdx6 protein used in the present study but in most mammalian species, including rat, mouse and bovine Prdx6; C47 is the lone Cys residue [6]. The conserved peroxidatic Cys (C47) also is the only Cys group that is present in recombinant human C91S-Prdx6 protein. The observed monoisotopic mass for both Prdx6 and C91S-Prdx6 in Tris buffered saline (TBS) deviated by −2 Da from the predicted monoisotopic mass of the sulfhydryl (Figure 2A), as we have published previously [26]. These results are consistent with oxidation of the protein to the sulfenic form, followed by the loss of H_2_O and formation of a sulfenylamide between the reactive Cys47 and an adjacent amino acid residue, possibly Thr48. In the presence of TCEP, the sulfenylamide bond is reduced, yielding proteins with the expected monoisotopic mass for Prdx6 and for C91S-Prdx6 (sulfhydryl forms). 

In the presence of GSH added to Prdx6 in TBS solution, the molecular mass of the protein increased to 24,920, a gain of 34 Da (Figure 2B). Our interpretation of this result is that the addition of GSH leads to reversal of the sulfenylamide to reform the sulfenic (+18 mass increase) and the subsequent oxidation of the sulfenic to sulfinic with the addition of an oxygen atom (+16 Da mass increase) accounts for the total increase in mass. A similar increase in mass of 34 Da in the presence of GSH was seen for C91S-Prdx6, indicating that the non-conserved Cys91 residue does not participate in the reactions, leading to the change in mass. Thus, the C47 residue in the Prdx6 proteins is paradoxically converted to a sulfinic acid (Cys-SOOH) in the presence of GSH; this sulfinic acid is not reduced by the subsequent addition of TCEP (Figure 2B). 

The formation of a sulfinic acid at Cys47 of Prdx6 was confirmed by alkylation of the cysteine residues in Prdx6 with iodoacetamide, followed by denaturation, reduction, and alkylation with acrylamide, trypsin digestion, and then analysis by liquid chromatography-tandem mass spectrometry (LC-MS/MS) in order to determine the levels of reduced, accessible cysteine (see Supplemental Figure S2). The presence of a sulfinic acid in the C47-containing digestion product of Prdx6 (i.e., the peptide DFTPVCTTELGR) was observed in the presence of GSH (with or without TCEP), but sulfinic acid was not observed in the peptide containing C91 (i.e., the peptide DINAYNCEEPTEK). 

### 3.4. GSH Induces Changes in Tryptophan Fluorescence and Circular Dichroism (CD) in hPrdx6

**Figure 3:**
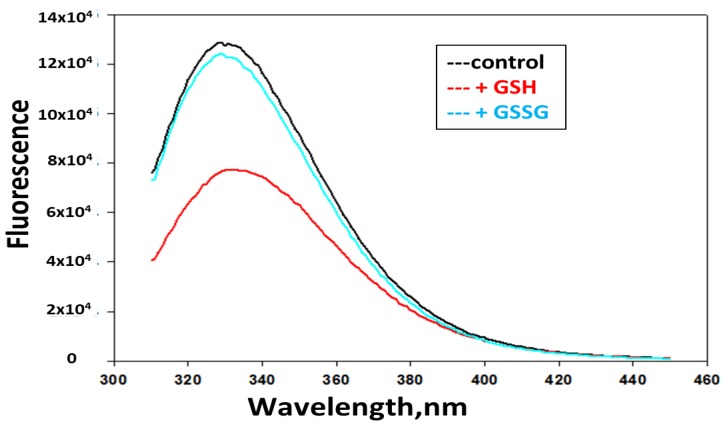
Effect of glutathione (GSH) and oxidized GSH (GSSG) on trptophan fluorescence emission of Prdx6 at pH 7. There is decreased fluorescence of Prdx6 in the presence of GSH while the presence of GSSG had no effect.

We examined potential changes in Prdx6 structure following the addition of GSH. Prdx6 has three tryptophan residues, two of which are in close proximity to the enzymatically active sites for peroxidase (C47) and PLA_2_ (S32) activities [12]. A change in tryptophan fluorescence is presumed to reflect a change in the local environment of tryptophan residues in the protein [29]. The addition of GSH resulted in a decrease of fluorescence intensity (Figure 3), which is compatible with the exposure of a tryptophan residue to a more polar environment [30]. There was no change in Trp fluorescence following the addition of GSSG. 

**Figure 4:**
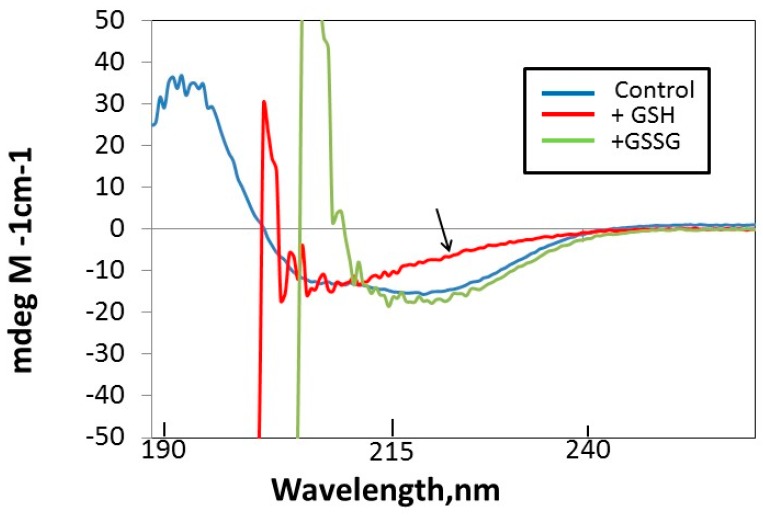
GSH-induced change in the secondary structure of Prdx6 as evaluated by far UV circular dichroism (CD). There is decreased (negative) molar ellipticity at 210–240 nm in the presence of GSH (arrow); there was no effect of GSSG at this wavelength.

The far UV circular dichroism (CD) profile of Prdx6 was also used to evaluate the effect of GSH addition on protein structure. Ignoring the noise below 215 nm, the results show that GSH, but not GSSG, induces a change in signal in the 210–240 nm region of the CD spectrum (Figure 4). This change reflects a decrease in the α-helical content of Prdx6. 

### 3.5. PLA_2_ Activity with Protein Modification

The PLA_2_ activity of Prdx6 at pH 7.4 was increased by the oxidation of the protein through its exposure to H_2_O_2_ (Table 1). This increase in activity at pH 7.4 with oxidized Prdx6 was about double the GSH-induced increase that was seen with non-oxidized Prdx6. Activity at pH 4 with oxidized Prdx6 was 102 ± 2 (*n* = 3), similar to the value at pH 7.4. There was no effect of GSH on aiPLA_2_ activity with either the oxidized protein or the oxidized substrate at either pH.

As we have shown previously, phosphorylation of Prdx6 also results in a marked increase in its aiPLA_2_ activity [22]. The increase in activity at pH 4 was ~20-fold and, fold-wise, was considerably greater at pH 7.4 (Table 1), so that the resultant activities of phosphorylated Prdx6 were similar at acidic and basic pHs [21,23]. This increased activity with Prdx6 phosphorylation greatly exceeded the increase in activity with Prdx6 oxidation.

## 4. Discussion

The major finding of the present study was that the addition of GSH to the medium that was used for assay of the PLA_2_ activity of Prdx6 resulted in: a) a physical change of protein structure, as shown by tryptophan fluorescence and far UV circular dichroism; b) the irreversible oxidation of the protein to the sulfinic acid (as indicated by MS analysis); and, c) an increase of its PLA_2_ activity at neutral pH. The increased aiPLA_2_ activity in the presence of GSH was inhibited by MJ33, a mimic of the enzymatic transition state for some PLA_2_ enzymes and a known inhibitor of aiPLA_2_ activity [31,32]. These results provide evidence that the catalytic mechanisms for stimulated PLA_2_ activity at pH 7 and basal activity at pH 4 are similar. Stimulation aiPLA_2_ activity at pH 7 appeared to be specific for GSH, since other sulfhydryls (GSSG, DTT, TCEP) were ineffective. Based on these results that confirmed a previous report [24], we conclude that GSH exerts its stimulatory effect on aiPLA_2_ activity through oxidation of the protein (Prdx6). 

Although this effect of GSH to promote Prdx6 oxidation initially was surprising, since GSH is a mild reductant, further consideration led us to propose the following mechanism. Based on the MS analysis, recombinant Prdx6 after purification and storage was oxidized to the sulfenylamide, a form that has been shown to “protect” some proteins against further auto-oxidation [33]; in the case of Prdx6, the auto-oxidizable site is C47, the active site for peroxidase activity. The Prdx6 sulfenylamide is formed by the oxidation of the thiol to the sulfenyl that rapidly reacts with an adjacent amine in the protein to form the stable sulfenylamide [25]; the specific amino group that was involved in this reaction has not yet been determined. The mechanism for auto-oxidation has been shown to reflect primarily the presence of trace metals [28,34,35]. In the presence of TCEP, the sulfenylamide state of the protein can be reversed first to the sulfenic and then reduced to the sulfhydryl; auto-oxidation of the protein under these conditions is prevented by the presence of the strong reductant (TCEP). GSH also is able to reverse sulfenylamide formation, as shown in previous studies with the enzyme protein tyrosine phosphatase B1 (PTPB1) [36]. This reversal reaction likely proceeds through the formation of a disulfide between GSH and the sulfenylamide to form a glutathionylated protein followed by hydrolysis to regenerate the sulfenyl [28]. However, GSH is a relatively weak reductant that cannot, by itself, reduce the sulfenic [3,10], and therefore cannot protect the protein against further oxidation to the sulfinic (and possibly sulfonic) form. Under physiological conditions *in vivo*, catalysis by glutathione S-transferase (GST) in the presence of GSH regenerates the sulfhydryl, thereby preventing its irreversible oxidation [10,37,38]. However, with severe oxidative stress or with GST deficiency, oxidation of Prdx6 could occur *in vivo,* resulting in a significant increase of intracellular aiPLA_2_ activity. It is important to note that this increase in aiPLA_2_ activity would be accompanied by loss of the GSH peroxidase activity of Prdx6 due to irreversible inactivation of the catalytic Cys 47.

**Figure 5:**
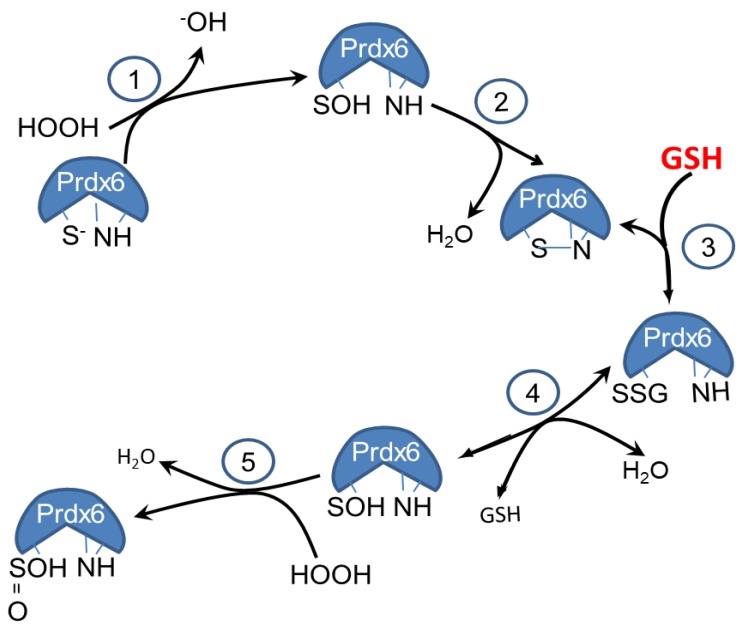
Reactions of Prdx6 with GSH *in vitro*. Reactions 1 and 2 occur during isolation of Prdx6 in the absence of GSH and GST; auto-oxidation of the protein by oxidant generation (shown here as HOOH) in the presence of trace metals leads to the formation of the Prdx6 sulfenic acid (reaction 1) and then dehydration to form the sulfenylamide (reaction 2). Reaction 2 is rapid when compared to the relatively slow rate of H_2_O_2_ formation (reaction 5 in the absence of GSH). Reactions 3 and 4 occur *in vitro* following the addition of GSH in the absence of GST. The sulfenylamide is reversed through glutathionylation of the protein (reaction 3), followed by reformation of the sulfenic acid (reaction 4). The rate of H_2_O_2_ generation is greatly enhanced in the presence of GSH [34], resulting in the oxidation of the sulfenic to the sulfinic (reaction 5 in the presence of GSH).

The reactions of Prdx6 that occur *in vitro* in the absence of GST are shown in Figure 5. To summarize these reactions, exposure of the protein to air during the isolation and storage of recombinant Prdx6 results in the oxidation of the thiol to an unstable sulfenylated protein that rapidly dehydrates to a stable sulfenylamide (reactions 1 and 2). The oxidant (shown as H_2_O_2_ in the figure) is generated from atmospheric O_2_ in the presence of trace metals [27,34,35]. Following the addition of GSH, there is regeneration of the sulfenic protein (reactions 3 and 4). In the presence of GSH, the rate of H_2_O_2_ generation through metal-catalyzed auto-oxidation is greatly increased [34], and the sulfenic can be oxidized to the sulfinic form (reaction 5). The irreversible formation of the sulfinic in the presence of GSH pulls the reaction cycle toward oxidation. Thus, the end result is Prdx6 over-oxidation following the addition of GSH to the protein *in vitro*, in the absence of GST. Under physiologic conditions in the presence of cellular GST plus GSH, the heterodimerization of Prdx6 with GST leads to a reduction of Prdx6 to the native sulfhydryl (reactions not shown) [26].

Similarly to the effect of GSH addition to the assay medium, oxidation of the protein by treatment with H_2_O_2_ prior to the assay also resulted in increased PLA_2_ activity (Table 1). A similar result was reported previously that used mutagenesis to show that the increase of activity with H_2_O_2_ treatment required the presence of C47 in Prdx6 [24]. aiPLA_2_ activity in the present study also was increased in the presence of a peroxidized lipid substrate (PLPCOOH), confirming our previous report [23]. Thus, several different reactions demonstrate a similar mechanism to stimulate aiPLA_2_ activity *in vitro*, namely the oxidation of the conserved Cys of Prdx6. These oxidative reactions include: (1) the presence of GSH; (2) pretreatment of the enzyme with H_2_O_2_; or, (3) use of PLPCOOH as the liposomal substrate. Each of these assay conditions in the absence of GST would be expected to result in the oxidation of the C47 of Prdx6. 

These findings reflect on the catalytic cycle for peroxidase activity. The reaction of H_2_O_2_ or a hydroperoxide substrate with Prdx6 will result in the formation of a sulfenic acid intermediate as the first step in the reduction of the oxidized lipid through peroxidase catalysis. In the absence of GST, the reaction cycle cannot be completed and the sulfenyl protein is susceptible to further oxidation, as shown previously using specific antibodies for the over-oxidized state of Prdx6 [23]. The presence of GST plus GSH prevents over-oxidation and it returns Prdx6 to its native sulhydryl form, thereby completing the peroxidase reaction cycle and preserving its peroxidase function.

Nevertheless, how does Cys oxidation affect aiPLA_2_ activity since the C47 residue in Prdx6, while being crucial for its peroxidatic function, has no direct role in the PLA_2_ reaction? Although our previous studies have shown that reduced (non-oxidized) phospholipid substrate does not bind at pH 7, it does bind to Prdx6 at pH 4; on the other hand, oxidized phospholipids bind to Prdx6 equally at acidic and neutral pH [23]. C47 is present as a thiolate at pH 7, but it is protonated to a sulfhydryl at pH 4, possibly resulting in changes in structure that facilitate the binding of substrate [2]. These binding data directly correlate with aiPLA_2_ activity. Binding of reduced substrate at pH 4 results in PLA_2_ activity, while binding of an oxidized phospholipid at pH 7 can result in PLA_2_ as well as peroxidase activity.

Crystal structure analysis has provided some insights into substrate binding [39]. The crystal structure of the sulfenic protein shows that the active site for Prdx6 binding (and aiPLA_2_-mediated catalysis) is on the protein surface and it is connected to the peroxidatic site by a narrow pocket. We have postulated that the oxidized phospholipid substrate is positioned for activity by binding of the head group to the protein surface and the insertion of the oxidized *sn*-2 fatty acid chain inside of the pocket [12]. On the other hand, crystal structural analysis of oxidized (sulfinic) Prdx6 indicates almost 10 angstroms distance between the H26 and S32 sites that were proposed for phospholipid binding. Because of this relatively long distance, it was suggested that the phospholipid actually may bind to the flat surface across the PLA_2_ active site and the peroxidatic active site of the other monomer [40]. However, several factors may modify the protein structure and its interaction with substrate. For example, over-oxidation of C47 to the sulfinic alters the Prdx6 secondary structure, as shown by CD analysis in this report. Further, the oxidation of C47 results in decreased affinity for homodimerization of the protein, resulting in an increased tendency for monomer formation [26]. Finally, phosphorylation (at T177) may lead to structural changes that alter the distance between the crucial amino acids [22]. We have shown, while using a zero-length chemical cross-linking and homology methodology, that several regions of reduced human PRDX6 are in a substantially different conformation from that shown for the crystal structure of the peroxidase catalytic intermediate [41]. Thus, the protein shows considerable plasticity and it can change conformation depending on many variables that could affect Prdx6-substrate interactions. The variable states of Prdx6 require further study before making definitive conclusions concerning the site and mechanism for binding of the phospholipid substrate to the enzyme.

## 5. Conclusions

Isolated recombinant human Prdx6 undergoing auto-oxidation to the sulfenic can form a sulfenylamide, a protected state that prevents auto-oxidation. The addition of GSH results in the regeneration of the sulfenic form that can be irreversibly oxidized to the sulfinic by oxidants that are produced through metal catalyzed auto-oxidation; the generation of oxidants through auto-oxidation occurs at a significantly increased rate in the presence of GSH. Over-oxidation of Prdx6 to the sulfinic results in a marked increase of its aiPLA_2_ activity at neutral pH, as shown previously [24] and in this manuscript. 

## Figures and Tables

**Table 1:** Effect of protein and substrate modifications on phospholipase A_2_ (PLA_2_) activity of peroxiredoxin 6 (Prdx6) at pH 7.4.

Condition	PLA_2_ Activity at pH 7.4 nmol/min/mg	
	−GSH	+GSH
Control	0.3 ± 0.06 (6)	51 ± 1 (6)
+MJ33	___	19 ± 2* (3)
+BEL	___	50 ± 1 (4)
+GSSG	3.2 ± 2.1 (3)	__
+DTT	2.0 ± 1.4 (3)	__
+TCEP	0.5 ± 0.1 (3)	__
Anaerobic	0.2 ± 0.1 (4)	0.2 ± 0.1* (4)
Liposomes with PLPC^†^	0.3 ± 0.02 (6)	48 ± 1 (6)
Oxidized liposomes with PLPC^†^	100 ± 1* (3)	100 ± 1* (3)
Phosphorylated Prdx6	1220 ± 6* (3)	1150 ± 3* (3)
Oxidized Prdx6	100 ± 3* (3)	99 ± 1* (3)

Human Prdx6 (2 µg) was pre-incubated with reagents for 15 min and then PLA2 activity was measured at pH 7.4 in Ca^2+^ free buffer. Substrate was 3H-1,2-Bis palmitoyl-sn-glycero-3-phosphocholine (DPPC) in mixed unilamellar liposomes. When added, glutathione (GSH) and other sulfhydryls were at 5 mM, MJ33 at 3 mol% of lipid, and BEL at 0.1 mM. Prdx6 was oxidized with H2O2 or was phosphorylated with Erk2. Values are mean ± SE for the number of experiments indicated in parentheses. * Significantly different (*p* < 0.05) from corresponding control (plus or minus GSH). † Liposomes with 1-palmitoyl, 2-linoleoyl, sn-glycero-3-phosphocholine (PLPC) replacing egg phosphatidylcholine (PC).

## Supplementary Materials

The following are available online at http://www.mdpi.com/2076-3921/8/1/4/s1, Figure S1: Purification of codon optimized recombinant human Prdx6 with an ion-exchange (DEAE cellulose) column, Figure S2: Relative quantitation based on the intensity of modified peptides from LC-MS/MS data analysis after treatment with GSH (+/−TCEP).
