# Supplementary material for: Oxidation of Peroxiredoxin 6 in the Presence of GSH Increases its Phospholipase A2 Activity at Cytoplasmic pH
Source: Antioxidants (Basel). 2018 Dec 24;8(1):4. doi: 10.3390/antiox8010004 (PMC6357108; doi:10.3390/antiox8010004)
Supplement: Supplementary file 1 [file antioxidants-08-00004-s001.pdf]

Supplemental data

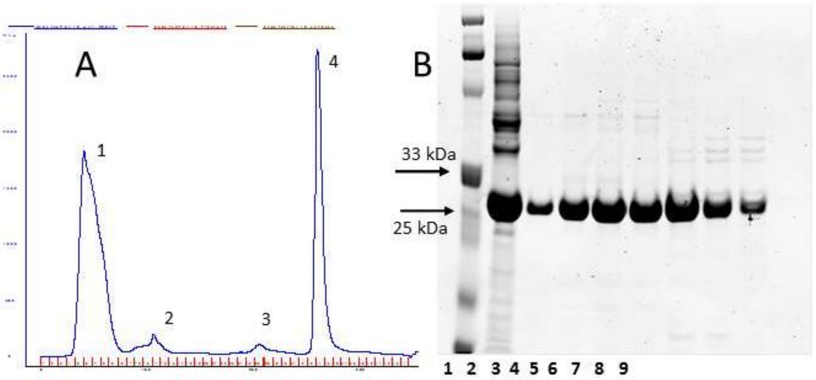

Figure S1. Purification of codon optimized recombinant human Prdx6 with an ion-exchange (DEAE cellulose) column. **A**. Analysis of effluent from the column.Peak 1,flow through (Prdx6); peaks 2 & 3, not analyzed; peak 4, proteins eluted with high salt. **B**. PAGE (stained with Coomassie blue) of the flow-through from the column. Lane 1:molecular weight markers; the arrow indicates 33 kDa; Lane 2: Starting material (input); Lanes 3–9: Fractions 8–14 from peak 1. The bands at ~25 kDa represent 97-99% of the Coomassie-stained protein in the lane.

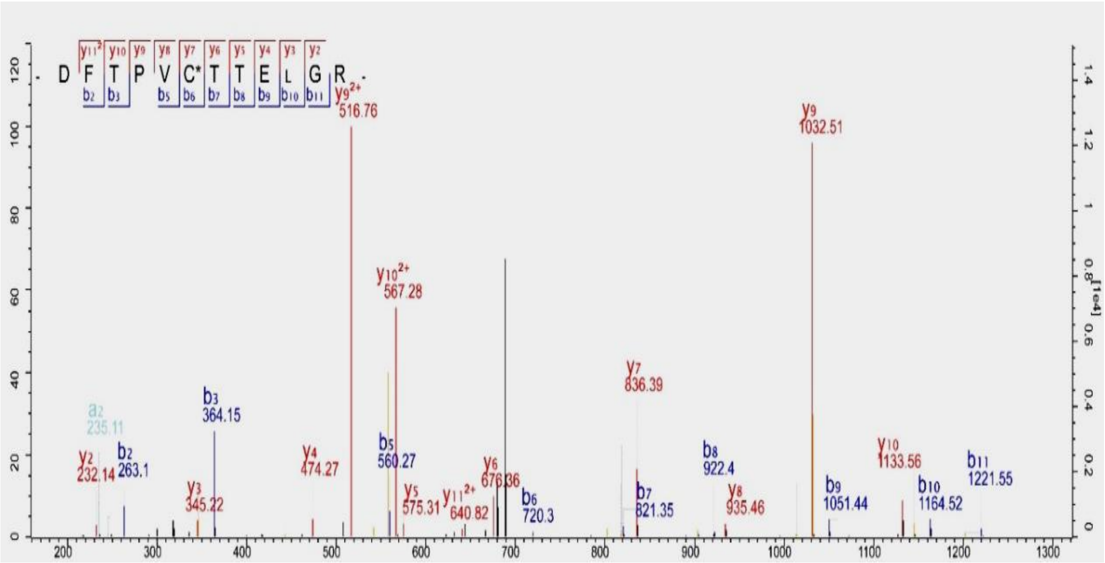

(A)

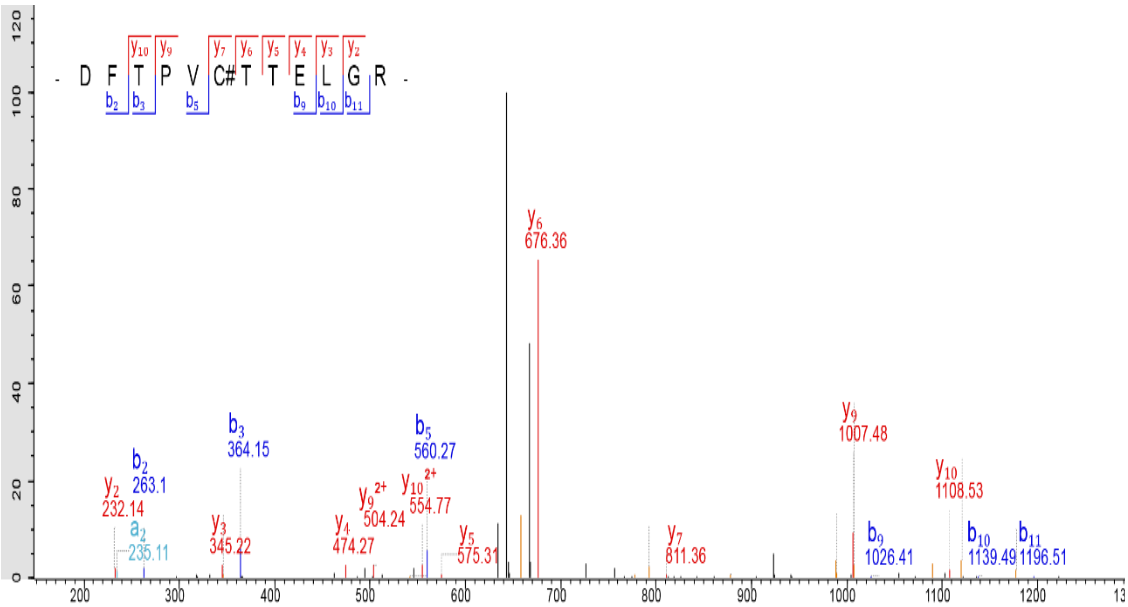

(B)

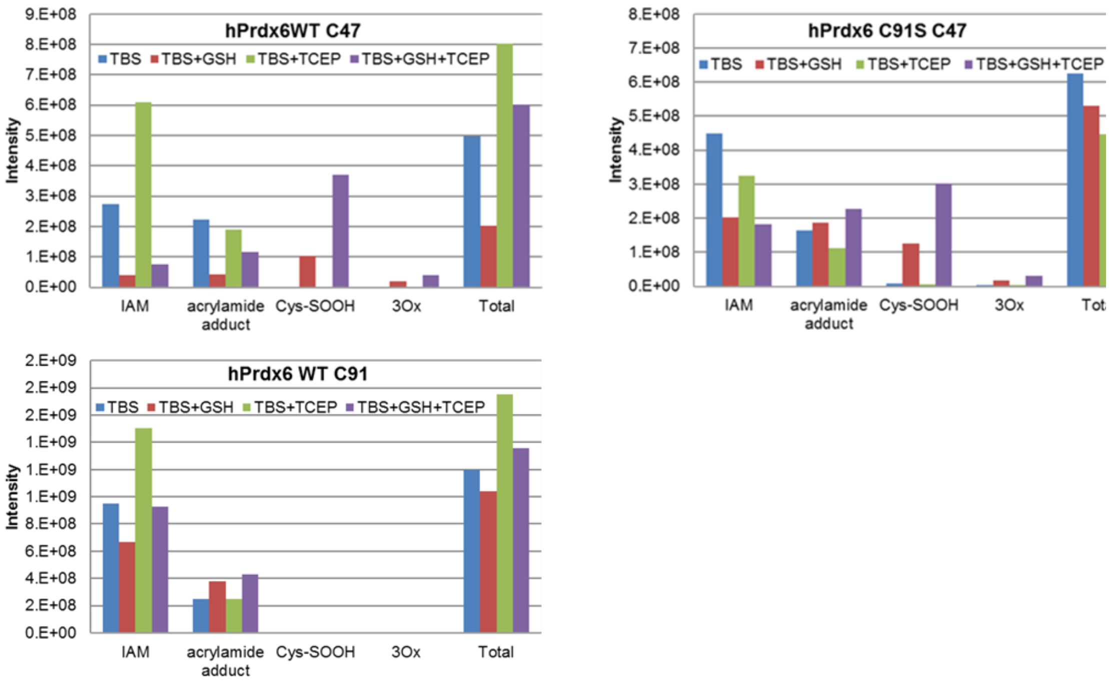

(C)

Figure S2. Relative quantitation based on the intensity of modified peptides from LC-MS/MS data analysis after treatment with GSH (+/- TCEP). **A**. LC-MS/MS fragmentation pattern of untreated hPrdx6WT (C*-iodacetamide derivative) **B**. LC-MS/MS fragmentation pattern of hPrdx6WT after treatment with GSH and TCEP (C#-Cys-SOOH) **C**. Intensity of the variable cysteine modifications on peptides C47 DFTPVCTTELGR and C91 DINAYNCEEPTEK in hPrdx6WT and hPrdx6C91S.
